# αD-Conotoxins in Species of the Eastern Pacific: The Case of *Conus princeps* from Mexico

**DOI:** 10.3390/toxins11070405

**Published:** 2019-07-12

**Authors:** Arisaí C. Hernández-Sámano, Andrés Falcón, Fernando Zamudio, César V.F. Batista, Jesús Emilio Michel-Morfín, Víctor Landa-Jaime, Estuardo López-Vera, Michael C. Jeziorski, Manuel B. Aguilar

**Affiliations:** 1 Laboratorio de Neurofarmacología Marina, Departamento de Neurobiología Celular y Molecular, Instituto de Neurobiología, Universidad Nacional Autónoma de México, Juriquilla, Querétaro 76230, Mexico; 2 Departamento de Medicina Molecular y Bioprocesos, Instituto de Biotecnología, Universidad Nacional Autónoma de México, Cuernavaca, Morelos 62210, Mexico; 3 Laboratorio Universitario de Proteómica, Instituto de Biotecnología, Universidad Nacional Autónoma de México, Cuernavaca, Morelos 62210, Mexico; 4 Departamento de Estudios para el Desarrollo Sustentable de Zonas Costeras, CUCSUR-Universidad de Guadalajara, San Patricio-Melaque, Jalisco 48980, Mexico; 5 Laboratorio de Toxinología Marina, Unidad Académica de Ecología y Biodiversidad Acuática, Instituto de Ciencias del Mar y Limnología, Universidad Nacional Autónoma de México, Ciudad de México 04510, Mexico; 6 Unidad de Proteogenómica, Instituto de Neurobiología, Universidad Nacional Autónoma de México, Juriquilla, Querétaro 76230, Mexico

**Keywords:** Cone snail, *Conus princeps*, αD-conotoxin, nAChR, hα7 nAChR, hα3β2 nAChR

## Abstract

*Conus* snails produce venoms containing numerous peptides such as the α-conotoxins (α-CTXs), which are well-known nicotinic acetylcholine receptor (nAChR) antagonists. Thirty-eight chromatographic fractions from *Conus princeps* venom extract were isolated by RP-HPLC. The biological activities of 37 fractions (0.07 µg/µL) were assayed by two-electrode voltage clamp on human α7 nAChRs expressed in *Xenopus laevis* oocytes. Fractions F7 and F16 notably inhibited the response elicited by acetylcholine by 52.7 ± 15.2% and 59.6 ± 2.5%, respectively. Fraction F7 was purified, and an active peptide (F7-3) was isolated. Using a combination of Edman degradation, mass spectrometry, and RNASeq, we determined the sequence of peptide F7-3: AVKKTCIRSTOGSNWGRCCLTKMCHTLCCARSDCTCVYRSGKGHGCSCTS, with one hydroxyproline (O) and a free C-terminus. The average mass of this peptide, 10,735.54 Da, indicates that it is a homodimer of identical subunits, with 10 disulfide bonds in total. This peptide is clearly similar to αD-CTXs from species of the Indo-Pacific. Therefore, we called it αD-PiXXA. This toxin slowly and reversibly inhibited the ACh-induced response of the hα7 nAChR subtype, with an IC_50_ of 6.2 μM, and it does not affect the hα3β2 subtype at 6.5 μM.

## 1. Introduction

The nicotinic acetylcholine receptors (nAChRs) play important roles in neuronal signaling. They modulate the release of neurotransmitters, such as dopamine, norepinephrine, γ-amino butyric acid, and acetylcholine (ACh). Therefore, they are implicated in a variety of pathophysiologies [1]. ACh is released from the pre-synaptic terminal, and then it binds to the extracellular domain of post-synaptic nAChRs, which orthosterically leads to the opening of the transmembrane channel to mediate a cationic current [2]. Thus, the discovery or development of selective compounds that target different subtypes of nAChRs could yield novel molecular tools, and also provide additional therapeutic agents or leads for the treatment of neurological disorders [3]. The homomeric α7 nAChR is one of the predominant nAChR subtypes in the central nervous system. This nAChR subtype is mainly distributed in the hippocampus and the cerebral cortex, regions associated with learning and memory mechanisms [3]. Major human pathologies such as epilepsy, schizophrenia, myasthenic syndrome, and Parkinson’s and Alzheimer’s diseases are associated with a dysfunction of α7 nAChRs, among other factors [3]. The α7 nAChR is also capable of inducing downstream signaling mechanisms in non-neuronal cells and is thought to be an ancestral form evolved in lower organisms that do not rely on fast excitatory mechanisms [4]. In humans, another common and predominant nAChR subtype is the α3β2 nAChR, which is expressed in the cerebellum, dorsal root ganglia, and spinal cord, and is involved in pain sensation [3,5].

Marine snails belonging to the genus *Conus* are venomous species distributed throughout tropical and subtropical waters. To date, more than 800 species of cone snails have been described, with more species likely remaining to be discovered [6]. They use fast-acting, and often paralyzing, venoms that are usually injected (small volumes, approximately ≤ 50 µL) into their prey or predator through a hypodermic needle-like modified radula tooth [4]. Molecular and phylogenetic studies have demonstrated that the evolution of envenomation strategies is typically a predatory rather than a defensive adaptation, but both are used by cone snails [7]. *Conus* venoms are complex mixtures of biologically active peptides. Mass spectrometry techniques have revealed that each species of cone snail produces from 200 to 1100, or even more, distinct toxins, termed conotoxins (CTXs), which may be neuroactive [8,9]. Typically, CTXs are small disulfide-rich peptides, between 10 and 40 residues in length (<5 kDa), and containing two to four disulfide bonds, that act in synergy to induce the rapid immobilization of preys or deterrence of predators [3]. Disulfide bridges provide exceptional structural stability, enabling a tight interaction with their molecular targets, mainly ion voltage- or ligand-gated channels, transporters, and G-protein-coupled receptors [9]. CTXs are usually potent and selective, and their size is an advantage for cost-effective synthesis and makes them ideal neuropharmacological probes [3,10].

Most of the CTXs that antagonize nAChR subtypes belong to the A-superfamily and they are known as alpha-conotoxins (α-CTXs) [11]. α-CTXs usually present a type I cysteine framework (CC-C-C), or eventually a type IV (CC-C-C-C-C). However, CTXs targeting nAChRs have been found outside the A superfamily, including nine other superfamilies (B3, D, J, L, M, O1, S, T, and a yet unspecified family), with at least seven more cysteine frameworks [3]. Besides their well-known value as pharmacological tools and receptor labels, proposed applications of α-CTXs include their use as pesticides, diagnostics, and therapeutics. Consequently, several α-CTXs have been studied for these purposes [12]. Recently, a novel type of CTXs affecting nAChRs has been identified, the αD-CTXs. These toxins occur naturally as dimers of identical or different monomers (47–50 residues) with complex disulfide connections (10 disulfide bonds per dimer) and possessing varying levels of posttranslational heterogeneity associated with proline and glutamic acid conversion to hydroxyproline and γ-carboxyglutamic acid, respectively [2,13]. αD-CTXs block α7 and α3β2 [13] and α9α10 [2] nAChRs.

α-CTXs from *Conus princeps* have been identified from their coding nucleotide sequences and some of them probably target α7 nAChR [14]. Therefore, our aim was to identify α-CTXs from *C. princeps* venom targeting human α7 nAChRs, to purify one of them, and to characterize it chemically and electrophysiologically. However, during the biochemical and electrophysiological characterization, we discovered the first αD-CTX from a non-Indo-Pacific species, because of its apparent selectivity over human α7 nAChR, it has potential for both neuroscience research and clinical applications.

## 2. Results

### 2.1. Peptidic Fractions of the C. princeps Venom and Determination of Activity on nAChRs

**Figure 1:**
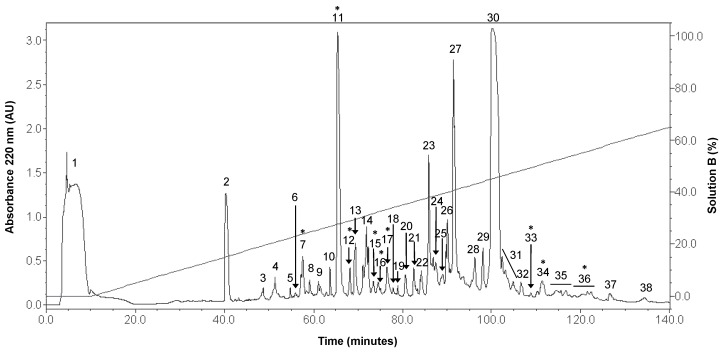
Fractionation by RP-HPLC of the *C. princeps* venom duct extract. An isocratic step at 0% Solution B for 10 min, followed by a linear gradient of 0% to 100% (*v*/*v*) of Solution B, over 200 min was used at 1 mL/min. The absorbance was measured at 220 nm. * Active fractions on human α7 nicotinic acetylcholine receptor (nAChR) inhibiting ≥25.0% the acetylcholine (ACh)-induced response.

**Figure 2:**
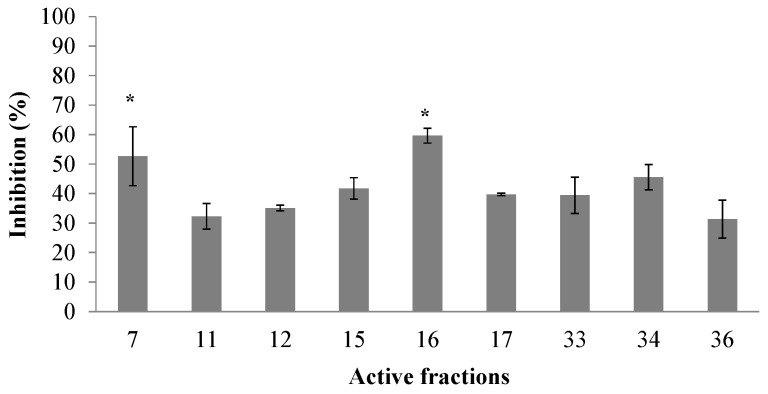
Active peptidic fractions (at 0.07 µg/µL) from *C. princeps* venom on human α7 nAChRs. Experiments were performed at least 3 times and the values averaged. *Active fractions on hα7 nAChRs inhibiting ≥ 50.0% the ACh-induced response.

Thirty-eight fractions were obtained from the crude venom duct extract of *C. princeps* by RP-HPLC (Figure 1), and the total protein obtained from this extract was 6.65 mg. All fractions, except the first one, were assayed at 0.07 µg/µL, by the electrophysiological technique of two-electrode voltage clamp in *Xenopus laevis* oocytes. We identified nine active fractions that inhibited by ≥25.0% the ACh-induced response of the human α7 subtype, including two fractions, named F7 and F16, which inhibited this response by more than 50.0% (Figure 2). 

**Figure 3:**
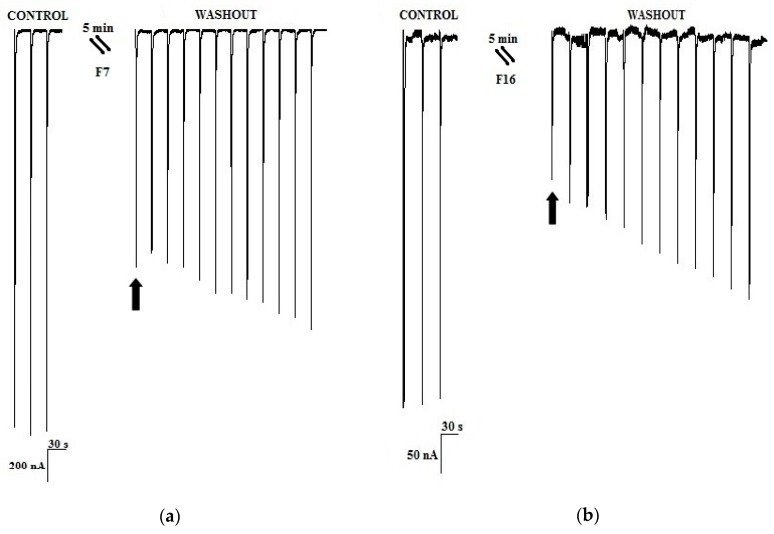
Representative current traces displaying the effect of active peptidic fractions (at 0.07 µg/µL) from *C. princeps* venom on human α7 nAChRs. (**a**) F7 fraction; (**b**) F16 fraction.

Fractions F7 and F16 represented 2.2% and 1.0% of protein, respectively, of the extracted venom from *C. princeps*. Fraction F7 blocked the ACh-induced response by 52.7 ± 15.2% (Figure 3a), while fraction F16 blocked 59.6 ± 2.5% (Figure 3b), and both effects were slowly reversible. Fraction F7 was obtained in sufficient quantity for further purification and analysis, but this was not the case for fraction F16.

### 2.2. Purification of an Active Peptide of C. princeps Venom and Determination of Activity on nAChRs

**Figure 4:**
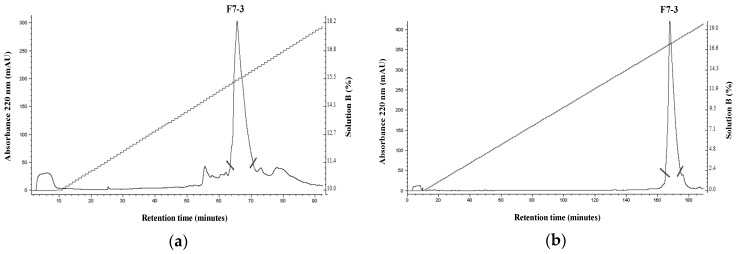
Purification of an active peptide of *C. princeps* venom by RP-HPLC. (**a**) F7 fraction: after an isocratic step at 10% Solution B for 10 min, followed by a linear gradient of 10% to 30% (*v*/*v*) of Solution B, over 200 min, at 1 mL/min was used. (**b**) F7-3 peptide: after an isocratic step at 0% Solution B for 10 min, followed by a linear gradient of 0% to 20% (*v*/*v*) of Solution B, over 200 min, at 0.8 mL/min was used. The absorbance was measured at 220 nm. Only the F7-3 peptide collected between the diagonal lines was used for further analyses.

**Figure 5:**
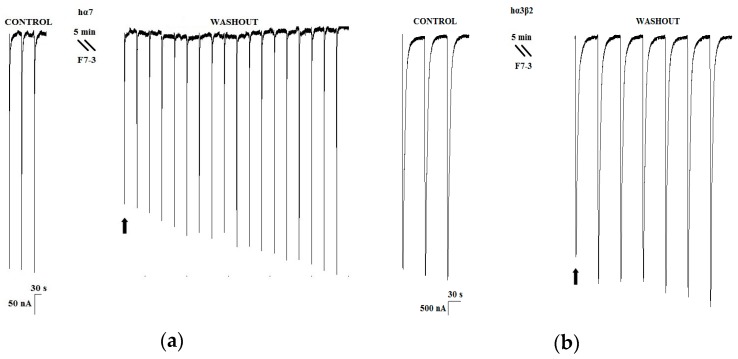
Representative current traces displaying the effect of the purified F7-3 peptide (at 0.07 µg/µL) of *C. princeps* venom on two human nAChR subtypes. (**a**) hα7 (*n* = 4); and (**b**) hα3β2 (*n* = 3).

**Figure 6:**
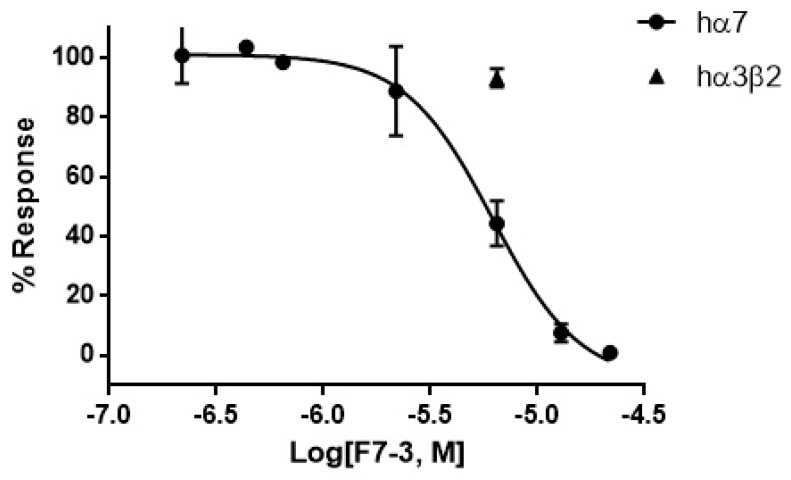
Concentration-response curve for the inhibition of human α7 nAChRs (circles) expressed in *Xenopus laevis* oocytes by the purified F7-3 peptide of *C. princeps* venom. The IC_50_ is 6.2 µM. The effect over human α3β2 (triangles) at 6.5 μM is also shown.

As mentioned above, within the nine identified active peptidic fractions of the *C. princeps* venom, fraction F7 was selected, due to its favorable combination of potency and relative abundance, for further purification by RP-HPLC (Figure 4a). The biological activity of the two-step-purified active peptide, named F7-3 (Figure 4b), was assessed once more by two-electrode voltage clamp recordings in oocytes. F7-3 peptide (at 0.07 µg/µL) inhibited 55.4 ± 15.2% the ACh-induced response on hα7 nAChRs and its effect was slowly reversible, returning to the initial activity 21 min after (only 18 ACh-pulses shown in Figure 5a). In contrast, at the same concentration, this peptide did not significantly inhibit the ACh-induced response on hα3β2 nAChRs (*n* = 3, 6.7 ± 3.1%) (Figure 5b). Therefore, we determined the half-maximal inhibitory concentration (IC_50_) on hα7 nAChRs, which was found to be 6.2 μM (95% confidence interval: 4.6 μM–8.2 μM) (Figure 6).

### 2.3. Molecular Mass and Amino Acid Sequence of the Purified Peptide

**Figure 7:**
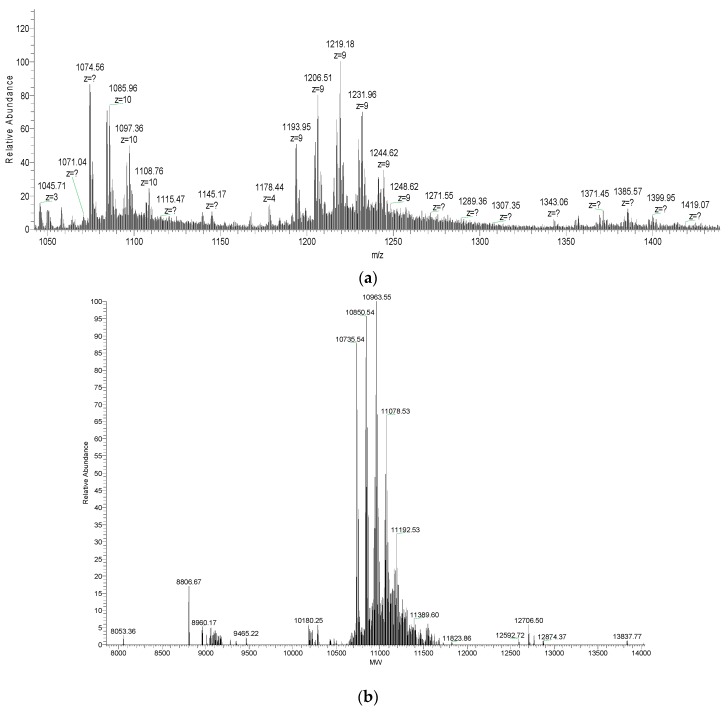
(**a**) Original mass spectrum of the native purified F7-3 peptide from *C. princeps* venom, the m/z signal (assuming z = 10) at 1074.56 corresponds to an average mass of 10,735.50 Da, which agrees very well with the m/z signal (z = 9) at 1193.95 which corresponds to an average mass of 10,735.54 Da. “?” denotes that the charge of the ion was not automatically determined by the software. (**b**) Deconvoluted mass spectrum of the native purified F7-3 peptide from *C. princeps* venom.

The molecular mass of the purified F7-3 peptide was determined by ESI-MS without reduction and alkylation. A series of major m/z average signals of 1085.96, 1097.36, and 1108.76 (z = 10) was obtained; also, another series of major average m/z signals with z = 9 was observed: 1193.95, 1206.51, 1219.18, 1231.96, 1244.62, and 1248.62 (Figure 7a). When deconvoluted, these signals correspond to average masses of 10,735.54, 10,850.54, 10,963.55, 11,078.53, and 11,192.53 Da (Figure 7b). The lowest mass suggests that F7-3 peptide is composed of ~98 amino acid residues, assuming an average residue mass of 110 Da. The difference between any pair of consecutive masses (~114 Da) indicates that, with the exception of the first species, the other are trifluoroacetic acid adducts of this species, as was first observed by Loughnan et al. [13].

**Figure 8:**
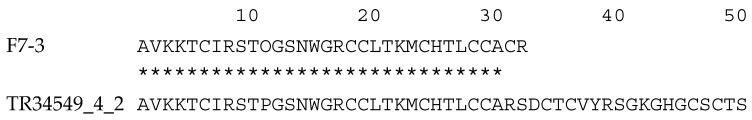
Top line, sequence of the purified F7-3 (αD-PiXXA) peptide from *C. princeps* obtained by automated Edman degradation; O is hydroxy-Pro. Bottom line, sequence TR34549_4_2 from the transcriptome of *C. princeps* venom duct; * indicates identical residues at the same position.

By automated Edman degradation, a 32-residue sequence was obtained (Figure 8). Hydroxy-Pro (O) was present at position 11. At positions 6, 18, 19, 24, 28, 29, and 31, blank cycles (“X”) were observed that likely correspond to Cys residues. Given this latter assumption, the theoretical average mass of this sequence is 3542.25 Da, according to Peptide Mass Calculator of the Mass Spectrometry and Biotechnology Resource of IonSourcesm mass spectrometry educational resource website [15], considering three disulfide bonds, one free Cys, one hydroxy-Pro residue, and a free C-terminus. This value is 7193.29 Da lower than the average mass of the native molecule (10,735.54 Da), which indicates that the sequence was not completely determined by ~66 residues.

The BLASTP similarity search revealed 25 significant hits (E value ≤ 1 × 10^−4^) to the F7-3 toxin; except for “conopeptide Mi039 [Conus miles]” the distinct annotations of these hits identify them as αD-conotoxins (data not shown). These toxins are covalent dimers of identical or similar polypeptide chains of ~50 residues containing 10 disulfide bonds in total [2,13,16,17], with two disulfide bonds linking the monomers [2].

Thus, we decided to compare the partial sequence of toxin F7-3, now renamed to αD-PiXXA, to the predicted mature α-D-conotoxin identified in the *C. princeps* venom duct transcriptome (transcript TR34549_4_2) [18] (to be published). It should be mentioned that the identities of the three last residues identified by automated Edman degradation are not totally reliable, due to the extremely low yield after 30–32 cycles. This comparison revealed a perfect match for 30 out of the 32 residues chemically identified, up to position 30 (Figure 8). This result strongly suggests that the sequence identified by RNASeq codes for that of the purified toxin.

Therefore, to verify this, we calculated the theoretical average mass of the sequence identified by RNASeq, considering one hydroxy-Pro residue for each monomer (directly observed by chemical sequencing of the purified F7-3 toxin), a free C-termini (deduced from the C-terminal sequence of the precursor identified by RNASeq) [18], and assuming 10 disulfide bonds (as the data from other researchers indicate [2,13,16,17]. This calculation yielded a value of 10,736.48 Da, which is in very good agreement with the experimental average mass of the intact toxin (10,735.54 Da). Thus, we conclude that we have established the complete sequence of toxin αD-PiXXA, which consists of a homodimer of two 50-residue chains, each containing a hydroxy-Pro residue and ten disulfide bridges in total [2].

## 3. Discussion

This work continues the study of the venom components of *Conus princeps*, a worm-hunter of the Eastern Pacific, from which one toxin, γ-CTX PiVIIA, has been recently purified and characterized at the chemical and electrophysiological levels [19,20]. Other toxins from this species have been identified by RT-PCR and transcriptomics [14,18,21]. Considering that most of the αA-CTXs identified by RT-PCR from *C. princeps* are predicted to target α7 nAChRs (but also α3β2 and other neuronal subtypes) [14], and the potential implications of α7 nAChRs antagonists to treat cancer, and cardiac and renal disorders [22], in this study we aimed to detect, purify, and characterize, toxins with activity at human α7 nAChRs from the venom of this species. Our identification of this activity in 24.3% (nine out of 37) of the venom fractions suggests a relevant role of α-CTX targeting an α7-like subtype in predators and/or prey.

There are reports on short (containing four Cys residues) αA-CTXs inhibiting human α7 nAChRs from molluscivorous and piscivorous *Conus* species, for example, α-PnIA and α-PeIA of *C. pennaceus* and *C. pergrandis*, respectively [23,24,25]. In the case of worm-hunting species, there are short αA-CTXs that inhibit the human α7 subtype, such as α-ImI, α-ImII, α-RegIIA, and α-LsIA [26,27,28], but there is also one long toxin belonging to the D-superfamily that inhibits human α7 nAChRs: αD-GeXXA [2].

The use of human α7 nAChRs in biological screening assays is relevant due to the potential application of α-CTXs as potential drugs, or leads for them, for human disease treatments [22]. In this sense, it should be pointed out that studies using both rat and human nAChRs have been revealed important differences in selectivity of some α-CTXs for several subtypes of these receptors [3]. For example, Yu et al. [29] compared the effects of α-CTX analogues [K11A]TxIB and [H5D]RegIIA over rat and human α7 nAChRs and found that they are much more potent on the rat than on the human receptor. However, it should be pointed out that other studies have shown differences between human and rat subtypes α9/α10 (for toxins RgIA [30] and Vc1.1 [31]) and α3β2 (for toxin RegIIA and its more potent analog [N11A, N12A]-RegIIA [32]). Interestingly, in all these cases, the affinity is higher for the rat subtypes, which has essential implications on the extrapolation of results obtained with rat nAChRs for applications related not only to the study of basic questions on the function of α7 nAChRs, but also to the treatment of human diseases.

As mentioned above, there are only two reports on α-CTXs from *C. princeps* at the protein level and they refer to the same peptide with activity over two different molecular targets [19,20]. Therefore, in this work, we isolated two venom duct extract fractions (F7 and F16) that inhibited ≥50.0% the ACh-induced response of the hα7 nAChR subtype, and purified and characterized the least scarce of the two most potent fractions. The purified peptide (F7-3) significantly inhibited the ACh-induced response of the hα7 subtype with a very slowly reversible effect and an IC_50_ = 6.2 μM, whereas it did not significantly affect the hα3β2 at 6.5 μM, which suggests that, regarding these two subtypes, it can be considered to be selective for hα7 nAChRs. However (please, see below), it might also affect the hα9/α10.

The amino acid sequence of this toxin (partially determined by chemical sequencing, but confirmed by RNAseq and mass spectrometry), unexpectedly, but undoubtedly, revealed that it is an αD-CTX, and, indeed, the first αD-CTX isolated from a species of the Eastern Pacific, αD-PiXXA. 

The short and long α-CTXs previously mentioned (PnIA, ImI, ImII, RegIIA, LsIA, and GeXXA) inhibit the hα7 subtype with higher affinity than αD-PiXXA [2,23,24,27,28]. However, an interesting feature of the αD-CTX characterized in the present work is that it dissociates from the receptor significantly more slowly (~21 min after starting the washout procedure, Figure 5) than most of the other toxins, with the exception of α-CTX LsIA (15 min after [28]).

A few αD-CTXs from species of the Indo-Pacific (all of them vermivorous) have been characterized at the electrophysiological level: αD-VxXIIB (currently named αD-VxXXB) from *Conus vexillum* [13], αD-Ms from *Conus mustelinus*, and αD-Cp from *Conus capitaneus* [17], inhibit the α7 subtype with the highest affinity (EC_50_), but also inhibit α3β2 and other nAChRs subtypes with clearly lower affinities. Nevertheless, it should be pointed out that the effects of all these toxins have been assessed only over rat subtypes. Interestingly, αD-GeXXA from *C. generalis* has been shown to inhibit the human α9/α10 subtype with higher affinity than the human α7 subtype [2]. Although the effect over the rat α9α10 subtype was highest among six rat (α9α10, α3β2, α3β4, α1β1δε, α4β2, and α4β4) and two human (α9α10 and α7) subtypes, the effect over the rat α7 subtype was not determined [2]. Therefore, this opens the question that other αD-conotoxins might have higher affinity for α9α10 than for α7 (from human and/or rat). Unfortunately, due to the availability of natural peptide, this question could not be answered for αD-PiXXA (this work), and it was not addressed for toxins αD-VxXXB [13], and αD-Ms and αD-Cp [17].

According to the most recent molecular phylogeny of cone snails [33], αD-conotoxins have been characterized from species belonging only to six worm-hunting *Conus* subgenera: *Rhizoconus* (*vexillum*, *capitaneus*, *mustelinus*, *miles*, and *rattus*) [13,17,34,35], *Strategoconus* (*vitulinus*, *generalis*, *planorbis*) [2,16,35], *Elisaconus* (*litteratus*), *Stephanoconus* (*imperialis*), *Splinoconus* (*tribblei*) [35], and *Ductoconus* (*princeps*; this work). Interestingly, αD-conotoxins were not detected in any of the four piscivorous and two molluscivorous species studied by RT-PCR [34]. However, it should be pointed out that they were not detected in species later shown to express them, such as *Strategoconus generalis* [2], *Stephanoconus imperialis*, *Elisaconus litteratus*, and *Rhizoconus miles* [35]. The subgenera in which αD-conotoxins have been identified are early- (*Stephanoconus* and *Strategoconus*), intermediate- (*Ductoconus*, *Splinoconus*, and *Rhizoconus*) and late-diverging (*Elisaconus*) among the *Conus* genus [33], which indicates that αD-CTXs are important members of the cone snails arsenal that have episodically evolved in this genus, although the possibility that they might be products of evolutionary convergence cannot be discarded [35]. However, the high sequence identity among the whole precursors (signal peptide, propeptide, and mature toxin) of the αD-conotoxins characterized so far (Figure 8B), suggests that convergence is not likely. Although the αD-conotoxins from the worm-hunters of the Indo-Pacific are all clearly major components of venom duct extracts [2,13,17] and have been demonstrated to have an important defensive role which has expedited the simplification of the defensive venom of *Rhizoconus* species [35], in *Conus princeps* αD-PiXXA is a minor component of venom duct extract (Figure 1), which suggests that in this species other types of conotoxins play important roles in defense. Nevertheless, the low abundance of PiXXA in the venom duct extract does not necessarily mean that it is not abundant in the injected venom and, therefore, it might be important for the defensive interactions of this species.

**Figure 9:**
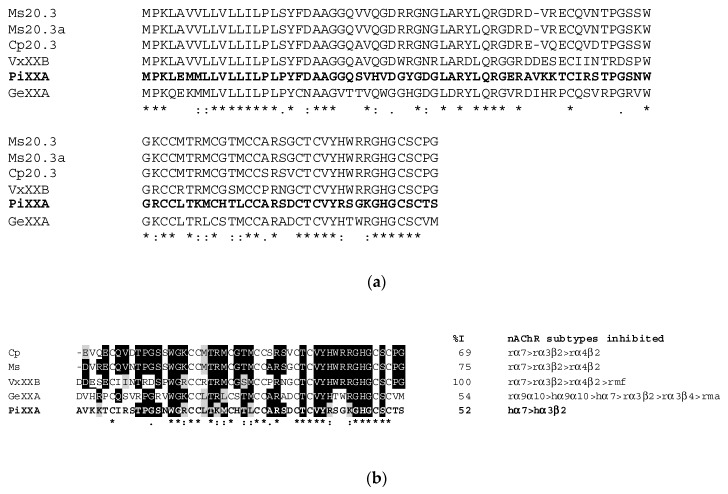
(**a**) Sequences of the precursors of PiXXA and of the alphaD-conotoxins whose biological activity has been demonstrated experimentally. (**b**) Comparison of the αD-CTX that have been characterized electrophysiologically. %I, percent identity with respect to the first αD-CTX studied at this level, αD-VxXXB [13]. The prefixes “r” and “h” stand for rat and human, respectively. nAChRs: mf, α1β1γδ; ma, α1β1δε. Cp and Ms, αD-Cp (major sequence) and αD-Ms, respectively [17]. GeXXA, αD-GeXXA [2], PiXXA (bold face), αD-PiXXA [this work]. Post-translational modifications: O, hydroxy-Pro; γ, gamma-carboxyglutamic acid. In both panels, positions with identical residues (*) and conserved (:) or semiconserved (.) substitutions are indicated at the bottom of the alignments. In panel B, with the exception of Cys, identical residues present in two or more sequences are highlighted by black background when at least one conserved substitution (highlighted by gray background) is present at the same position.

In order to gain insight into the structure-activity relationships of these infrequent, dimeric, vermivorous toxins, we compared the sequences of the αD-CTXs that have been characterized at the electrophysiological level. The multiple alignments of PiXXA with VxXXB, Ms, Cp, and GeXXA (Figure 9) clearly shows that the toxins of the species belonging to the *Rhizoconus* subgenus (*vexillum*, *capitaneus*, and *mustelinus*) are more related to each other than to those from both *Strategoconus* (*generalis*) and *Ductoconus* (*princeps*). This is not unexpected and has also been observed by Prashanth et al. in a study including species belonging to the *Stephanoconus*, *Strategoconus*, *Splinoconus*, and *Elisaconus* subgenera in a phylogenetic tree including toxins from six *Rhizoconus* species [35].

αD-GeXXA is the one toxin that has been tested over α9α10 nAChRs (rat and human) and shown to be more potent over this subtype than over α7 (human) [2]. Despite αD-GeXXA has some residues that are not present in any of the αD-CTX from the *Rhizoconus* species (even as conserved substitutions), such as Val-2, His-3, Asp-33, and Thr-40 (and that shares, as such or as conserved substitutions, with αD-PiXXA), we cannot point to any of these residues as responsible for the highest affinity of αD-GeXXA for the α9α10 subtype. As mentioned above, the amount of natural toxin prevented testing αD-PiXXA over this subtype.

In summary, this work is the first report of αD-CTXs from species of the Eastern Pacific, specifically from the vermivore *C. princeps* from Mexican coasts. Like the αD-CTXs from species of the Indo-Pacific (also vermivorous), αD-PiXXA has a clear antagonistic effect over α7 nAChRs (human). Due to their remarkably different three-dimensional structure with respect to other nAChR-affecting toxins and their allosteric mechanism of action, αD-conotoxins are expected to contribute to our growing knowledge of ligand interactions with nAChRs [2]. The isolation of αD-CTXs targeting neuronal hα7 nAChRs from barely studied species such as *C. princeps* could provide novel molecular tools and therapeutic agents, or leads for rationally designing them, for the treatment of a range of neurological disorders that involve these receptors [22].

## 4. Materials and Methods

### 4.1. Reagents

Genetic engineering products: Wizard Plus SV Minipreps DNA Purification System was purchased from Promega Corp. (Madison, WI, USA); Not I restriction enzyme from Thermo Fisher Scientific (Waltham, MA, USA), mMESSAGE mMACHINE kit for high yield capped RNA transcription from Ambion Inc. (Austin, TX, USA); QIAquick PCR Purification and RNeasy Mini kits from Qiagen (Hilden, Germany). Gentamycin was obtained from Life Technologies Co. (Grand Island, NY, USA). High Performance Liquid Chromatography (HPLC) reagents: Trifluoroacetic acid (TFA) was acquired from Thermo Scientific (Rockford, IL, USA); HPLC-grade acetonitrile (ACN) from Sigma Chemical Co. (St. Louis, MO, USA). Bradford protein assay kit from Thermo Scientific (Rockford, IL, USA): oxidized insulin B chain, bovine serum albumin (BSA), type I collagenase, and acetylcholine (ACh) from Sigma Chemical Co. (St. Louis, MO, USA). All other reagents were analytical grade.

### 4.2. Obtainment of Crude Venom Extract

*C. princeps* specimens were captured off the coasts of the Mexican Eastern Pacific Ocean (state of Jalisco) and conserved at −70 °C. The venom ducts of 35 specimens were isolated and homogenized in 4.0 mL of 40% (*v*/*v*) ACN in water containing 2.0% (*v*/*v*) TFA. The homogenate was centrifuged at 10,000 g for 15 min, and the crude venom extract (supernatant) used for fractionation by HPLC as described below. Protein content was analyzed by the Bradford method using BSA as standard [36].

### 4.3. Fractionation of the Crude Venom Extract 

Aliquots (1.5 mg of protein/534 μL) of the obtained crude venom extract were centrifuged at 10,000 g for 15 min and the supernatant was taken to 5 mL with Solution A (aqueous solution with 0.1% (*v*/*v*) TFA) before of the fractionation by Reversed Phase-High Performance Liquid Chromatography (RP-HPLC) in a Waters 600 HPLC System (Milford, MA, USA) using a Vydac Peptide & Protein C18 column (218TP54, 5 µm particle size, 4.6 mm i.d. × 250 mm). The column was equilibrated at room temperature with Solution A. Fractions of the crude venom extract were eluted with a linear gradient from 0% to 100% (*v*/*v*) of Solution B (90% ACN in water containing 0.085% (*v*/*v*) TFA), over 200 min, after an isocratic step of 0% Solution B for 10 min, at a flow rate of 1 mL/min. The elution profile was monitored at 220 nm. Fractions were quantified by comparison of areas yielded by the HPLC system in comparison with a standard curve of oxidized bovine insulin B chain.

### 4.4. Purification of the Active Peptide

The first step of purification was performed by RP-HPLC using a linear gradient from 10% to 30% (*v*/*v*) of Solution B, over 200 min, after an isocratic step at 10% Solution B for 10 min at a flow rate of 1.0 mL/min. A second step of purification was done using a linear gradient from 0% to 20% (*v*/*v*) of Solution B, over 200 min, after an isocratic step at 0% Solution B for 10 min at a flow rate of 0.8 mL/min in the same HPLC system. The elution profile was monitored at 220 nm. Fractions were quantified as described above for the crude venom extract. 

### 4.5. Determination of Electrophysiological Activity on nAChRs

Ovarian lobes from *Xenopus laevis* frogs were removed and placed in OR-2 solution (82.5 mM NaCl, 2.0 mM KCl, 1.0 mM MgCl_2_∙6 H_2_O, and 5 mM HEPES, pH 7.3); then they were defolliculated with 1.25 mg/mL type 1 collagenase in OR-2 solution for 40 min at room temperature and slowly stired. The *Xenopus* oocytes were then washed five times with 5 mL of OR-2 solution. Stage VI oocytes were selected and incubated in ND-96 (96.0 mM NaCl, 2.0 mM KCl, 1.0 mM MgCl_2_∙6 H_2_O, 1.8 mM CaCl_2_∙2 H_2_O, 5 mM HEPES, pH 7.3)/gentamycin (8 µg/mL) solution, at 15 °C. Oocytes were injected 1 day after harvesting.

cDNA clones encoding neuronal human α3 and α7 and β2 nAChR subunits were kindly provided by Dr. J. Michael McIntosh (Department of Psychiatry and Department of Biology, University of Utah, Salt Lake City, UT, USA). After amplification, purification, linearization, and purification of linearized cDNAs, cRNA was obtained using the in vitro RNA transcription kit, according to the protocol of the manufacturer. cRNAs were purified and confirmed on 1.5% agarose/buffer MOPS (MOPS-sodium acetate-EDTA, pH 7) gels, and stored at −80 °C. For expression of neuronal human α7 and α3β2 at 55.8 ng and 13.3 ng of the corresponding cRNAs, respectively, were injected into each oocyte with a Drummond Nanoject II Auto-Nanoliter injector (Drummond Scientific, Broomall, PA, USA). Each injected oocyte was incubated in ND-96/gentamycin solution at 15 °C [37]. Oocyte recordings were obtained after 72 h through day six post-injection.

A two-electrode voltage clamp amplifier was operated (Oocyte Clamp OC-725C, Warner Instruments Corp., Hamden, CT, USA) to measure the effects of *C. princeps* chromatographic fractions on neuronal human α7 nAChRs expressed by *Xenopus laevis* oocytes and the purified peptide on hα7 and hα3β2 nAChRs [37]. Resistances were 2–3 megohm for voltage and 0.5−1.0 megohm for current electrodes. The membrane potential was clamped at −70 mV. The oocytes were kept in a chamber (volume ≈ 40 μL) with ND-96 solution at a constant rate of 1 mL/min. Three microlitres of the fraction or purified peptide (1.0 μg/μL) was directly pipetted into the static chamber for 5 min prior to exposure to 1-s pulses of 100 µM ACh in ND-96 solution. Three μg of chromatographic fractions or purified toxin was used for a final concentration of 0.07 μg/μL. The amount of sample was defined considering a peptide of approximately 3 kDa and molarity of 23.3 µM, in order to be able to detect toxins with low affinity for human nAChR. Data acquisition was automatically controlled by a home-made virtual instrument constructed with the graphical programming language LabView 8.6 (National Instruments, Austin, TX, USA). All recordings were made at room temperature. The average of seven control ACh-induced responses just preceding a test response was used to obtain the % inhibition. At least three oocytes were assessed in each assay (*n* ≥ 3).

The concentration-response curve for the purified toxin was determined with 0.1, 0.2, 0.3, 1.0, 3.0, 6.0, and 10.0 μg (which correspond to concentrations of 0.2, 0.4, 0.6, 2.2, 6.5, 13.0, and 21.7 μM). All electrophysiological data were pooled (*n* = 2−4 oocytes for each data point).

### 4.6. Determination of Molecular Mass and Amino Acid Sequence of the Purified Peptide

The molecular mass of one native purified peptide (F7-3, 2.0 µg dissolved in 10 µL of 0.1% aqueous formic acid) was determined by a LC-MS system composed by a nano-flow liquid chromatographer Dionex 3000 and a hybrid Orbitrap Velos mass spectrometer with nano-electrospray ion source, both from Thermo-Fisher (San Jose, CA, USA). Calibration of the mass spectrometer was carried out using Calmix solution consisting of 10 different calibrants allowing measurements with accuracies better than 5 parts per million. The nano-flow chromatographic system was operated at 350 nL/min, using an isocratic gradient of 50% Solvent B (0.1% acetic acid in ACN; Solvent A, 0.1% acetic acid in water) for 30 min. A micro-needle (New Objective, Woburn, MA, USA) was used in the nano-electrospray ionization source operating with 2.0 kV of spray voltage.

The amino acid sequence of the purified peptide was determined by automated Edman degradation. A PPSQ-31A Protein Sequencer (Shimadzu Scientific Instruments, Columbia, MD, USA) was used. The purified peptide (F7-3, 5 µg) was sequenced without reduction and alkylation; the sample was dissolved in 5 µL of 60% (*v*/*v*) aqueous ACN containing 1.0% acetic acid. An aliquot of 4 µL was taken and diluted with 10 µL of 37% aqueous ACN. The mixture was then loaded onto a TFA-treated glass fiber disk (073-04171, Wako Pure Chemical Industries, Ltd., Osaka, Japan) previously treated with polybrene (Sigma-Aldrich Corp., St. Louis, MO, USA) according to manufacturer’s protocol. The Glass Fiber Disk protocol was used for 32 cycles.

### 4.7. Similarity Search and Sequence Alignment

Protein-protein BLAST, BLASTP 2.9.0+ [38], was used to search the nr (Non-redundant protein sequences) database, with Organism = Conus (taxid:6490), and the default parameters.

Clustal W 2.1 [39] in the GenomeNet of the Kyoto University Bioinformatics Center [40] was used for multiple sequence alignment, using the default parameters, except that, in order to obtain the most accurate results, the option for Pairwise Alignment was changed from the default (FAST/APPROXIMATE) to SLOW/ACCURATE.

### 4.8. Statistical Analysis

All analyses were carried out at least in triplicate, mean values ± standard deviations were reported. Differences were statistically accepted at P < 0.05. The half-maximal inhibitory concentration (IC_50_) value was determined using Prism 2.0 (GraphPad Software, San Diego, CA, USA), by fitting the data obtained from the concentration-response relationship to the equation (1):

(1) $$R=R_{\min}+[(R_{\max}-R_{\min})/(1+10\log(IC_{50}-C))]$$

where *R* is the response (percent inhibition) at a given concentration (*C*) of αD-PiXXA, *R*_min_ is the minimum response, *R*_max_ is the maximum response, and *IC*_50_ is the concentration that produces 50% of *R*_max_. Standard errors of the mean were used for this calculation.
